# Diabetic ketoacidosis: knowledge and practice among patients with diabetes attending three specialized diabetes clinics in Khartoum, Sudan

**DOI:** 10.11604/pamj.2022.41.299.31129

**Published:** 2022-04-13

**Authors:** Almutasim Billah Elbagir Elhassan, Marwa Mohammed Elrasheed Saad, Mohammed Suliman Tawer Salman, Abazr Ibrahim, Almigdad Ali, Asmaa Abd Elaal Abd Alla, Fadwa Mohammed Saad

**Affiliations:** 1 Faculty of Medicine, University of Khartoum, P.O. box 102, Al Qasr Avenue, Khartoum 11111, Sudan

**Keywords:** Diabetic Ketoacidosis (DKA), knowledge, practice

## Abstract

**Introduction:**

diabetic Ketoacidosis is the leading cause of mortality in children and adolescents with T1DM and accounts for about 50% of all deaths in patients younger than 24 years with diabetes. It affects 8 out of 1000 people with diabetes annually, with a worldwide mortality rate of 2-10%. The purpose of this study is to assess the knowledge and practice towards DKA among patients with diabetes attending three diabetes clinics in Khartoum.

**Methods:**

we conducted a cross-sectional institution-based study. It included all patients with diabetes attending three diabetes clinics in Khartoum state over the period July – September 2016. A self-administered questionnaire was used to assess and score knowledge and practice towards DKA among patients with diabetes. Data were analyzed using SPSS 23.0.

**Results:**

one hundred and ten patients participated in the study, of whom 86 had heard of DKA and were questioned further. Fifty-six point nine percent of participants had poor knowledge (0-8 out of 24) and low practice scores (0-2 out of 6). There was a strong association between knowledge scores and attended clinics.

**Conclusion:**

there was an evident lack of knowledge and poor practice towards DKA among patients with diabetes attending diabetes clinics in Khartoum, Sudan.

## Introduction

Diabetes Mellitus is a disorder in which glucose is not oxidized to produce energy due to a lack of insulin. It is divided into two types: Type 1 Diabetes Mellitus (T1DM), where there is an absolute or near absolute deficiency of insulin, and Type 2 Diabetes Mellitus (T2DM), where there is resistance to insulin with an inadequate compensatory increase in insulin secretion [1]. According to the International Diabetes Federation, there are more than 19 million people with Diabetes in Africa, with an expected increase of 143% by 2045. It is estimated to have caused 366,200 deaths in 2019. Among those, 25,800 were children aged 0-19 diagnosed with T1DM [2]. One of the important complications of Diabetes Mellitus is Diabetic Ketoacidosis. Diabetic Ketoacidosis is a state of relative or absolute insulin deficiency aggravated by hyperglycemia, dehydration, and acidosis [3]. Numerous factors have been known to cause DKA. The most common causes include infections and treatment errors. For example, subtherapeutic insulin dose, omitted dose, failing to increase the dose during illness, and infusion set failures. Other causes include Gastrointestinal disease, Cardiovascular events, Inflammatory diseases, Pancreatitis, alcohol abuse, and drugs [4,5]. In Sudan, the most common precipitating factor is poor compliance [6]. It is predominantly associated with T1DM, but people with T2DM can experience Ketoacidosis during severe infections and other illnesses [7]. Three metabolic abnormalities exist simultaneously in DKA: hyperglycemia, hyperketonaemia, and metabolic acidosis [8]. Laboratory parameters used to diagnose DKA include; Blood glucose greater than 216mg/dl (12mmol/l), the presence of ketonuria, and an arterial blood pH of less than 7.35 [9]. Diabetic Ketoacidosis is the leading cause of mortality in children and adolescents with T1DM and constitutes about 50% of all deaths in patients with diabetes younger than 24 years of age [10,11]. It affects 8 in 1000 people with diabetes annually [12], with worldwide mortality of 2-10% [13]. In a study of 3,000 patients with T1DM, 9% were admitted with DKA one or more times over 12 months [14]. Diabetic Ketoacidosis remains the highest cause of death for patients with diabetes under 20 years of age [15]. Delays in presentation and treatment increase mortality.

Clinical features of DKA include dehydration, hypotension, tachycardia, hyperventilation, Kussmaul breathing, ketotic breath, hyperthermia, polyuria, and polydipsia due to osmotic diuresis, nausea and vomiting, weight loss, generalized weakness, abdominal pain, drowsiness, and coma [7]. In a study done in Saudi Arabia, in king Abdulaziz Hospital, almost all patients presented with abdominal pain and vomiting (83.3 and 91.7%, respectively). Dyspnoea was present in 28%, and fever in 31% of the patients. The underlying cause was insulin cessation in 86.6%, followed by infection at 38% [16]. There is a high rate of DKA in T1DM patients in the Arab world, as quantified by H Zayed in a systematic review. He found the rate of DKA to range from 17% in Egypt to 100% in Morocco, with an overall rate of 46.7% [17]. At the same time, most cases of DKA are preventable by effective self-management, thus reducing the incidence, morbidity, and mortality. Education can prevent 70% of DKA episodes, claims Jervell; therefore, patients need to be well informed about diabetes management to prevent further episodes of DKA [17]. An important aspect of the educational program that must be implemented is the Sick Day Rules, which include increasing fluid intake, increasing caloric intake, frequently testing blood glucose, urine ketones, and blood ketones, and taking extra insulin [18]. A study showed a 28.6% DKA incidence in children living in east and central Poland. The study recommended increasing awareness of symptoms of DKA among children below five years and those between 10-12 years, especially that the highest rate of DKA was noted in children younger than [19]. A Danish survey report of all 205 T1DM patients who were treated with subcutaneous insulin infusion (CSII) between 2006 and 2015 in the outpatient clinic of Frederica hospital showed an extremely low incidence of DKA (1 in 100 patients), all of which were due to infusion set failures. They attribute their low incidence to their education program on infusion set failures [5]. Existing literature on the knowledge, attitude, and practice of patients with diabetes towards diabetes and its complications showed different results in different countries and ethnic groups. Patients in Palestine and Ghana showed poor knowledge of diabetes and its complications [20,21]. While patients in Somalia showed good knowledge, but poor practice and attitude [22]. A study done in Glasgow compared knowledge, attitude, and practices regarding T2DM among patients of British, Indian, and Pakistani origin. The study found that the knowledge of ethnic groups about diabetes is poor [23]. There is a gap in Sudanese literature on the knowledge and practices of Sudanese patients with diabetes towards DKA. This study aims to bridge that gap and provide a reference for researchers.

## Methods

**Design of the study**: this was a cross-sectional institution-based study undertaken in three specialized diabetes clinics in Khartoum state, Sudan.

**Study setting**: this study was undertaken in Mohammed Ali Altoum´s center, Diabetes Clinic at Soba University Hospital, and Ahmed Gasim Pediatric hospital, spanning the three cities in Khartoum state; Omdurman, Bahri, and Khartoum. Mohammed Ali Altoum´s center clinic, located in Omdurman, is held every day except Fridays. It receives an average of 8-10 patients a day; half of those patients have diabetes, while the second half has other endocrinological diseases. The diabetes clinic in Soba university hospital, located in Khartoum, is held once a week, averaging 15-20 patients each clinic. Doctors provide health education in the clinic. The diabetes clinic at Ahmed Gasim pediatrics hospital, located in Bahri is held once weekly; 13-17 patients attend the clinic each week. Patients attending this clinic are usually provided with a diabetes self-care manual and a free glucometer.

**Study participants and sampling**: all patients with diabetes attending diabetes clinics at Soba University Hospital, Ahmed Gasim hospital, and Mohammed Ali Altoum centre during the study period from Jul 24^th^ to Sep 27^th^ 2016 participated in the study.

**Research tool design, data collection, scoring, and analysis**: data were collected using a self-administered structured questionnaire. The authors designed the questionnaire based on the Clinical Practice Guidelines and Standards of Care of Diabetes Mellitus in Sudan 2011 [24]. We also involved a social scientist and doctors specialized in community medicine in the design of the questionnaire. The questionnaire was tested on a small sample of the population (5 patients) prior to data collection. The questionnaire was designed to assess important aspects of knowledge and practice towards DKA and was split into three modules. Seven and three questions were used to evaluate the knowledge and practices, respectively. All questions were multiple-choice questions; participants were awarded one mark for each correct answer while wrong answers carried no mark. Overall, knowledge questions carried 24 marks. Scores were classified into three categories poor (0-8), average (9-16), and good >16. Practice questions carried six marks. Similarly, scores were classified into poor (0-2), average (3-4), and good >5. Data was entered into and analyzed using Statistical Package for the Social Sciences (SPSS) version 23. Chi-square test was used to measure the association between the knowledge grade and clinic attended, and a P value of <.05 was considered significant.

**Ethical clearance**: ethical approval was obtained from the department of community medicine, faculty of medicine, University of Khartoum, and ethical committees of hospitals and centers included in the study. Verbal informed consent was obtained from participants or their carer if aged less than 15 years.

## Results

**Socio-economic profile of respondents**: one hundred and ten patients participated in the study; 35 were male (32.8%), 75 were female (68.2%). The mean age was 34.6 ± 21.757 years, with the youngest being three years old and the oldest eighty years old. Thirty-two participants were under 15 years old; they had their questionnaires filled with assistance from their caregivers, who were aged between 20 and 70 years with a mean age of 35 years. Twenty-eight point one percent (28.1%) were male, and 71.9% were females. Socio-economic status of the participants is shown in Table 1.

**Table 1:** socio-economic status of participants

Gender of participants	Frequency (%)
Male	35(31.8%)
Female	75(68.2%)
**Total**	110
**Age of participants**	
Mean	34.63
Median	38.5
Standard deviation	21.757
**Education of participants**	
Illiterate	15(19.2%)
Khalwa(traditional religious education)	4(5.1%)
Primary school	29(37.2%)
High school	20(25.6%)
Graduates	10(12.8)
**Total**	78
**Education of caregivers**	
Illiterate (traditional religious education)	5(15.6%)
Khalwa	1(3.1%)
Primary school	8(25.0%)
High school	9(28.1%)
Graduates	9(28.1%)
**Profession of participants**	
Unskilled labour	5(6.4%)
Housewife	41(52.6%)
Employee	6(7.7%)
Driver	1(1.3%)
Health sector employee	2(2.6%)
Student	14(17.9%)
Free lancer	22(3.2%)
Retired	1(1.3%)
**Total**	78
**Profession of caregivers**	
Unskilled labour	2(6.3%)
Housewife	16(50.0%)
Unemployed	3(9.4%)
Student	3(9.4%)
Free lancer	3(9.4%)
Retired	1(3.1%)
Military	2(6.3%)
**Total**	32

**Knowledge of DKA**: eighty percent of participants did not know which type of diabetes they have, 14.5% reported having T1DM, and 4.5% reported having T2DM. Twenty-four (21.8%) participants have never heard of DKA. The remaining 86 (78.2%) were questioned on the causes, symptoms, complications, sick day rules, and blood sugar level in DKA (Table 2). Seventy-two percent had learned about DKA from a doctor, and twenty-seven percent learned about it from a friend or relative who suffered from it. Forty-nine (56.9%) participants had poor knowledge scores (0-8); they could not identify most of the causes, symptoms, complications, sick day rules, and blood sugar levels in DKA.

**Table 2:** number of participants who identified causes, signs & symptoms, complications, sick day rules, and sugar blood levels in DKA

Causes	Number of participants
Missed insulin dose	43 (39%)
Poor diet	47(42.7%)
Stress	17(15.5%)
Illness	39 ( 35.5%)
Heart attack	5(4.5%)
Certain medications	6(5.5%)
**Signs and symptoms**	
Polydipsia	58(52%)
Polyuria	54(49.1%)
Nausea and vomiting	35(31.8%)
Abdominal pain	35 (31.8%)
Breathlessness	23(20.9%)
Altered mental status	29(26.4%)
Sweat breath	14(12.7%)
Generalized weakness	56 (50.9%)
**Sick day rules**	
Increase insulin dose	50(45.5%)
Increase fluid intake	53(48.1%)
Monitor blood sugar	61(55.5%)
**Blood sugar levels in DKA**	
<100 mg/dl	2 (1.8%)
> 200 mg/dl	40(36.4%)
I do not know	68(61.8%)
**Complications**	
loss of consciousness	43 (39.1%)
Death	29(26.4%)

**Practices related to DKA**: various questions were used to assess the practices related to diabetes and the prevention of DKA. Forty-nine participants had an overall poor practice grade (0-2). Fifty-seven percent of participants owned a glucometer; however, only 13.1% measured blood glucose at the recommended rate. Thirty-nine participants reported having had a recent illness and were questioned further on their practices during the illness period, according to sick day rules (Table 3). There was a strong association (p <0.001) between knowledge grade achieved and hospital/center attended. Fifteen out of the nineteen patients with good knowledge scores attended Ahmed Gasim Paediatric Hospital. Forty-four point five percent (44.5%), 38.3%, and 17.3% of the participants scored poor, average, and good grades, respectively, in terms of knowledge. Forty-four point five percent (44.5%), 44.5%, and 10.9% of the participants scored poor, average, and good grades in terms of practice. Table 4 shows the distribution of the participants´ grades in different centers.

**Table 3:** practices of patients during illness period in percentages

Practice	Percentage of participants
Increased fluid intake	64.1%
Increase insulin dose	38.5%
Increase insulin measurement	82%

**Table 4:** the knowledge grade for each hospital/center

	Soba hospital	Ahmed Gasim paediatric hospital	Mohammed Ali altoum center	Total
Poor	29	9	13	49
Average	17	23	2	42
Good	4	15	0	19
**Total**	48	47	15	110

## Discussion

Eighty percent of patients did not know which type they had, and several mentioned that this was the first time they knew that there were two types of diabetes. This might be due to unfamiliarity with the terminology as the participants were able to describe the characteristics of each type of diabetes. Most participants had poor knowledge and practice towards DKA, although it is a life-threatening complication of diabetes, indicating insufficient health education on the topic. This can be partially due to the study including patients with T2DM, of which DKA is not a common complication. Knowledge of the two most common causes of DKA, infections and omitted doses, was poor, 35.5% and 39.1%, respectively. Similar to findings in Saudi Arabia, where 9% and 29% of participants related DKA to infection and febrile illness, and 32% of them recognized missed insulin dose as a cause [25]. These are alarming results, indicating that most patients are vulnerable to developing DKA in the future.

In our study, 21.8% of the respondents had never heard of DKA. This figure is lower than a study done in Saudi Arabia, where 33% stated they do not know what DKA is [26]. Seventy-two percent of our participants learned about DKA from a doctor, while 27% learned about it from a friend or a relative who suffered from DKA. In contrast, 48.6% and 41% of the participants in Othman study were educated about DKA by a diabetes care and education specialist, and a physician, respectively [26]. There were no diabetes care and education specialists available in the study area except in Mohammed Ali Altom center. The most frequently recognized symptoms of DKA were polydipsia and polyuria, 52% and 49%, respectively, while the knowledge of other symptoms was low. Prior knowledge of symptoms of DKA protected newly diagnosed T1DM pediatric patients from developing DKA in Barbara Davis Center in Aurora, Colorado. Where DKA incidence was significantly lower in families who were familiar with DKA symptoms prior to the diagnosis of their child, compared to families who were not [27]. Less than one-third identified death as a complication, underestimating the gravity of DKA. Overall, 44.5% of participants had a poor knowledge score, similar to 54% among adults with diabetes in KSA [25].

More than 50% of participants had a glucometer; however, only 13% measured blood sugar at the recommended rate. On the other hand, 87% of patients with diabetes in Karachi were measuring blood sugar at the recommended rate [28]. Challenges to frequent measurement were the unavailability and cost of glucometer strips. Almost all participants (93%) on insulin therapy took insulin regularly, showing good compliance. During periods of illness, most participants monitored their blood glucose regularly and increased their fluid intake, while only one-third of participants increased their insulin dose. This seems to be a common suboptimal practice. In the study done in Saudi Arabia, only 13% of patients with diabetes increased their insulin dose during illness [26]. Campbell *et al*. showed that people with diabetes with excellent practice (regular blood glucose monitoring, regular insulin injections and less frequently missed doses, varied insulin carbohydrate ratios for meals) had significantly lesser DKA prevalence (2%) than those with poor practice (21%) [29]. Our study found that most patients with good knowledge scores have attended a clinic that provided health education. (p<.001) There is evidence to support that health education reduces the incidence of DKA. In a study done in Australia, a diabetes awareness campaign has reduced the rate of DKA at the initial diagnosis of T1DM in children by 64% [30].

**Limitations**: this study was limited by small sample size, and the research tool could have been improved by psychometric testing.

## Conclusion

The researchers have found the knowledge and practice of patients with Diabetes attending diabetes clinics in Khartoum towards DKA to be very poor. There is a need to raise awareness of DKA causes, signs and symptoms, and prevention measures. This can be achieved through health education in clinics, as well as community campaigns.

### What is known about this topic

- The knowledge of patients with diabetes about Diabetes Mellitus and its complications varied globally and regionally, with good knowledge in the United Kingdom and Somalia and poor knowledge in Saudi Arabia, Palestine, and Ghana;
- Globally the most common precipitant of DKA is infections, while in Sudan, the most common cause is poor compliance.

### What this study adds

- More than half of Sudanese patients with diabetes (56.9%) attending specialized diabetes clinics in Khartoum had poor knowledge and practice towards Diabetic Ketoacidosis, which outlines a gap in knowledge and practice that tailored educational programs could target.
